# Bronchial thermoplasty: activations predict response

**DOI:** 10.1186/s12931-017-0617-7

**Published:** 2017-07-04

**Authors:** David Langton, Joy Sha, Alvin Ing, David Fielding, Francis Thien, Virginia Plummer

**Affiliations:** 1 Department of Thoracic Medicine, Frankston Hospital, 2 Hastings Road, Frankston, VIC 3199 Australia; 2 Faculty of Medicine, Nursing and Health Sciences, Monash University, Melbourne, VIC Australia; 3 Faculty of Medicine and Health Sciences, Macquarie University, Sydney, NSW Australia; 4 Department of Thoracic Medicine, Royal Brisbane and Women’s Hospital, Herston, QLD Australia; 5 Eastern Health Clinical School, Box Hill Hospital and Monash University, Melbourne, VIC Australia

**Keywords:** Severe asthma, Bronchial thermoplasty, Response predictors, Activations, Technique

## Abstract

**Background:**

Bronchial thermoplasty (BT) is an emerging bronchoscopic intervention for the treatment of severe asthma. The predictive factors for clinical response to BT are unknown. We examined the relationship between the number of radiofrequency activations applied and the treatment response observed.

**Methods:**

Data were collected from 24 consecutive cases treated at three Australian centres from June 2014 to March 2016. The baseline characteristics were collated along with the activations delivered. The primary response measure was change in the Asthma Control Questionnaire-5 (ACQ-5) score measured at 6 months post BT. The relationship between change in outcome parameters and the number of activations delivered was explored.

**Results:**

All patients met the ERS/ATS definition for severe asthma. At 6 months post treatment, mean ACQ-5 improved from 3.3 ± 1.1 to 1.5 ± 1.1, *p* < 0.001. The minimal clinically significant improvement in ACQ-5 of ≥0.5 was observed in 21 out of 24 patients. The only significant variable that differed between the 21 responders and the three non-responders was the number of activations delivered, with 139 ± 11 activations in the non-responders, compared to 221 ± 45 activations in the responders (*p* < 0.01). A significant inverse correlation was found between change in ACQ-5 score and the number of activations, *r* = −0.43 (*p* < 0.05).

**Conclusions:**

The number of activations delivered during BT has a role in determining clinical response to treatment.

## Background

Whilst most asthmatic patients can be well controlled using currently available inhaler therapy, there remains a small percentage of severely symptomatic patients whose daily lives are limited by their chronic condition [1]. Given the high prevalence of asthma worldwide, numerically this represents many millions of people with an unmet need for effective therapy [2].

In randomized clinical trials, bronchial thermoplasty (BT) has been shown to be an effective and safe additional modality for the management of patients with poorly controlled asthma despite standard therapy [3–5]. Typically, selected patients have daily symptoms, and frequent exacerbations requiring corticosteroids, despite high dose inhaled corticosteroids and long acting bronchodilators.

BT is performed during flexible bronchoscopy using a radiofrequency catheter to deliver thermal injury to airways between 3 and 10 mm in size [6]. Both animal and human studies have demonstrated that, as result, there is a reduction in airway smooth muscle mass in the areas treated, whilst the airway mucosa recovers undamaged [7–9]. Airway smooth muscle is typically hypertrophied in asthma, responsible for producing variable bronchoconstriction and hence wheezing [10]. Clinical studies have demonstrated that 12 months following treatment, significant improvements are achieved in patient symptom scores, reliever medication usage, and asthma exacerbations requiring prednisolone [3–5]. There is however very little data regarding the characteristics of those who fail to respond to BT, nor the contributing reasons.

It is possible that there is operator dependent variation in the adequacy of the radiofrequency treatment delivered to the airways during BT. If so, this could be responsible for a lack of response in some participants. Currently there is no routine clinical method of measuring airway smooth muscle thickness. However, a surrogate marker for treatment effectiveness can be found in the number of actuations delivered by the radiofrequency system. This measure is routinely available at every case, and is the total of the number of 10-s radiofrequency heat charges delivered to the airway. Whilst recommendations have been provided on the number of actuations that constitute effective treatment [11], there is an absence of existing evidence reporting on the relationship between activations and clinical outcomes. It is tempting to speculate that a lower number of actuations may reflect less airway smooth muscle treated, but this may also depend on patient factors such as airway size. Similarly, we cannot conclude whether higher numbers of total activations results in better asthma control, and if there is any impact on immediate post-procedure recovery.

Therefore, in this study, we examine retrospectively in our case series whether there is any relationship between number of radiofrequency actuations delivered and the patient response observed, as measured by improvement in symptom scores.

## Methods

### Study participants

Twenty four consecutive participants with severe, poorly controlled asthma were treated with BT between June 2014 and March 2016 at three Australian university teaching hospitals. All patients had been under the regular care of a specialist respiratory physician prior to referral to the treating proceduralist. Patients were chosen for bronchial thermoplasty at the discretion of the treating team. Subsequently, the medical records of each patient were reviewed and the baseline characteristics of the participants collated, including age, gender, height, weight, preventative and reliever asthma medication, smoking history, spirometry, and the Asthma Control Questionnaire Score (ACQ-5).

### Procedure

Each proceduralist was head of bronchoscopic services in their hospital and had at least 25 years experience in performing bronchoscopy. All proceduralists had been trained in using the Alair bronchial thermoplasty system (Alair, Boston Scientific, NSW, Australia), and used the Olympus BF Q190 (Olympus Australia, Victoria, Australia) bronchoscope under general anaesthesia. The cases reported were the first cases that the proceduralists had undertaken. BT was conducted in three treatments, 3 to 4 weeks apart, starting with the right lower lobe, then left lower lobe, and, at the last treatment, both upper lobes. Patients were treated with oral prednisolone for 3 days before and 3 days after the procedure. All patients were electively observed in hospital for 24-48 h post procedure, during which time they also received nebulised bronchodilators. The number of radiofrequency activations was recorded for each treatment session.

### Measurements

The primary outcome measure chosen was change in the ACQ-5 from baseline. This measure is known to exhibit stability over time and to be responsive to improvements in asthma symptoms [12]. It was administered by study nurses immediately prior to commencing BT, and again 6 months following the final bronchoscopy. These nurses were blinded to intraoperative care such as the number of activations administered. Spirometry was conducted in accredited respiratory laboratories by experienced scientific staff and according to European Respiratory Society/American Thoracic Society (ERS/ATS) standards [13]. For uniformity between sites, the predicted equations used were from Quanjer [14].

### Safety

An adverse event was recorded for any participant who required admission of longer than 48 h, or any participant who was readmitted to hospital for any cause within 30 days of any procedure.

### Ethics

Approval to collate and audit data as part of quality assurance was provided by the Human Research and Ethics Committee at each participating institution. Participants were assigned a unique study-specific identifier number, and clinical data was shared between institutions using these numbers so that no individually identifiable data was disclosed. Specific permission to use the ACQ-5 in this project was sought from, and granted by, the author Elizabeth Juniper. All participants provided informed consent to treatment and data collection.

### Statistical analysis

SPSS version 24 (IBM corporation, New York, USA) was used for all statistical analyses. Grouped data has been reported as mean ± standard deviation unless the data was not parametric, in which case median (Interquartile range(IQR)) was used. A paired t-test was undertaken to compare all paired sets of normally distributed data, whilst a Wilcoxon signed rank test was used for paired non-parametric data. Where the sample size was very small, the data was assumed to be non parametric and a Mann-Whitney U test was used. Statistical significance was taken as *p* < 0.05 for a two-tailed test. Pearson’s Correlation Coefficient was calculated for bivariate continuous normally distributed data, and univariate linear regression was performed if a significant relationship was observed. The mean radiofrequency treatment activations per patient at the three different participating institutions were compared by ANOVA. A multivariate linear regression model was created to examine clinical factors that might predict the number of activations delivered to an individual patient.

## Results

### Baseline characteristics

Twenty four patients, 16 females and 8 males, completed treatment and 6 months follow-up. The mean age was 55.4 ± 12.6 years (range: 27–75). The mean BMI was 28.8.8 ± 6.7 (range 22.2–46.9). 22 patients were never-smokers, whilst 2 were ex-smokers with a less than 10 pack-year history. The mean baseline prebronchodilator FEV_1_ was 61.8 ± 15.9% predicted (range: 33–95%); 12 cases (50%) had an FEV_1_ of <60% predicted, and six cases FEV_1_ < 50% predicted. The mean change in baseline FEV_1_ after administration of salbutamol was 13.7 ± 12.7% (range 0–46.7%).

All participants had been prescribed high doses of inhaled corticosteroids, mean beclomethasone equivalent dose of 2095 ± 450mcg daily (range: 1000-3000mcg). Twelve patients (50%) were taking maintenance oral prednisolone (median dose 10 mg/day, range 4-20 mg). All patients (100%) were taking long-acting beta_2_ agonists and long-acting muscarinic antagonists. Additional preventative therapy included leukotriene receptor antagonists (42%), omalizumab (29%), and methotrexate (17%).

Every patient met the ERS/ATS definition for severe asthma, by fulfilling at least one of the four criteria [1]. Symptom control remained poor despite the extensive preventative treatment. The mean ACQ-5 score was 3.3 ± 1.1. Patients used a median of eight salbutamol puffs per day for rescue reliever therapy (IQR 4–15) and, in the 6 months prior to treatment, there was a median of two exacerbations requiring prednisolone per patient (IQR 0–5).

### Response to treatment

Table 1 presents the comparison at 6 months post treatment with baseline for the main outcome parameters. Favourable responses to bronchial thermoplasty were seen in all parameters including FEV_1_.

**Table 1:** Response to BT treatment, *n* = 24

	Baseline	6 months post	*p*
ACQ-5	3.3 ± 1.1	1.5 ± 1.1	<0.001^a^
FEV_1_%predicted	61.8 ± 15.9	68.7 ± 15.6	<0.05^a^
Salbutamol puffs/day	8 (11)	2 (2)	<0.001^b^
Exacerbations/6months	2.0 (2.75)	0 (1)	<0.001^b^
PNL mg/d *n* = 12	10 (7.5)	0 (4.5)	<0.005^b^

*mean ± SD*, median (IQR), *PNL* prednisolone, ^a^paired t test, ^b^ Wilcoxon signed rank test

### Responder analysis

An improvement in ACQ-5 of greater than 0.5 units (the minimal clinically significant difference) [12] was observed in 21 of 24 participants (88%). The three non-responders were compared with their counterparts across a range of clinical variables, including age, gender, baseline FEV_1_%, baseline bronchodilator response, medication usage and exacerbation frequency (Table 2). The only significant difference between the two groups related to the number of radiofrequency treatments. The mean treatment in non-responders was 139 ± 11 activations, compared to 221 ± 45 activations in the responders, *p* < 0.01.

**Table 2:** Responder comparison

	Responders	Non-responders	*p*
n	21	3	-
Change in ACQ-5	−2.2 ± 1.0	+1.1 ± 1.0	-
RF activations	221 ± 45	139 ± 11.4	<0.01^a^
Age (yrs)	56.7 ± 12.7	47.0 ± 8.9	NS
Male gender	33%	33%	NS
BMI kg/m^2^	28.9 ± 7.0	28.1 ± 4.5	NS
Baseline FEV_1_%	61.2 ± 16.8	65.5 ± 8.2	NS
BD response%	13.3 ± 13.0	14.9 ± 8.5	NS
Salbutamol puffs/day	8 (12)	4 (5)	NS

*BD* bronchodilator response % change in FEV1, *NS* not significant, ^a^Mann-Whitney U

### Radiofrequency treatment

**Fig. 1:**
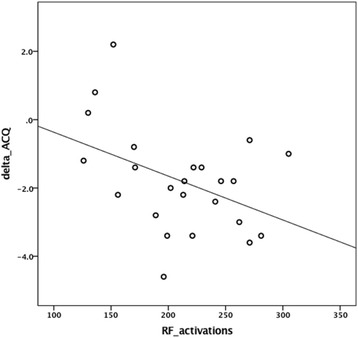
Radiofrequency activations versus change in ACQ-5 after BT

A mean of 211 ± 50 radiofrequency activations per patient (range: 121–305) were delivered. The relationship between activations delivered and treatment response measured by change in ACQ-5 was explored by correlation and regression analysis. This is presented graphically in Fig. 1. The Pearson correlation coefficient for this relationship is *r* = −0.43, *p* < 0.05. A P-P plot suggested that the data fitted most closely with a linear model. The regression line was given by the equation *ACQ delta = 0.92–0.01 x activations*, r^2^=0.18, *p* < 0.05. No correlation was observed between activations and change in FEV_1_%predicted (*r* = −0.1, *p* = 0.64).

### Technique

Technique related differences were observed between proceduralists. Significantly fewer radiofrequency activations were delivered by proceduralist A (155 ± 24, *n* = 8) by comparison with proceduralist B (235 ± 24, *n* = 10) or proceduralist C (244 ± 47, *n* = 6) (ANOVA, *p* < 0.001). Proceduralist A’s patient data were compared to those of Proceduralist B and C using an independent t-test, and the results are presented in Table 3. In an otherwise similar population of patients, the fewer activations by Proceduralist A may have reduced the magnitude of improvement in ACQ following BT.

**Table 3:** Results comparison by proceduralist

	Proceduralist A	Proceduralist B + C	*p*
n	8	16	
Activations	155 ± 24	241 ± 33	<0.001^a^
Change in ACQ-5	−0.9 ± 2.1	−2.3 ± 1.0	<0.05^a^
Change in FEV_1_%	9.8 ± 19.2	6.1 ± 15.1	NS
Age (yrs)	47.8 ± 10.7	59.1 ± 12.3	<0.05^a^
Male gender	50%	25%	NS
BMI kg/m^2^	26.1 ± 3.6	29.9 ± 7.8	NS
Baseline ACQ-5	3.0 ± 1.0	3.4 ± 1.1	NS
Baseline FEV_1_%	61.3 ± 9.0	62.3 ± 19.3	NS

^a^independent t-test

A multivariate linear regression model was created to examine the contribution of variables that might influence the number of radiofrequency activations delivered to each patient. The variables examined included: age, gender, height, weight, baseline FEV_1_% predicted and proceduralist. The only variable found to significantly influence activations was the proceduralist, and the effect was strong, *r* = 0.727, *p* < 0.001.

### Adverse events

In this series of 72 procedures, there were no deaths, and no readmissions for any cause within 30 days of a procedure. One case, on two occasions, required monitoring in Intensive Care immediately post procedure for an asthma exacerbation, and on one of these occasions received non-invasive ventilation before making a complete recovery. During the procedures, there were no instances of pneumothorax nor airway haemorrhage.

## Discussion

The patients in this study comprise a group of severe asthmatics - more severe than were treated in the two larger randomized control trials of bronchial thermoplasty [3, 4]. In the AIR and AIR2 trials, the mean prebronchodilator FEV_1_ was 72.7% and 77.8% predicted respectively, compared with 61.8% in the series we report. Furthermore, few patients were included in the above trials who required maintenance oral corticosteroids, monoclonal antibodies or immunosuppressants. However, the outcomes achieved across a broad range of parameters are superior to the AIR and AIR2 studies. Importantly, in this case series, a significant improvement in prebronchodilator FEV_1_ has been demonstrated following treatment - this being a missing feature of the larger clinical trials in BT. In this respect our series is similar to the RISA trial which also demonstrated a significant improvement in FEV_1_ with treatment. More severely obstructed patients it seems may have more to gain from BT.

This is the first study to have specifically examined the relationship between radiofrequency activations and clinical response. A significant relationship has been demonstrated between activations applied and improvement in ACQ-5. This is consistent with our understanding of the biological effect of BT on airway smooth muscle. However, two studies have examined the effect of BT on human airway smooth muscle obtained from endobronchial biopsies [7, 8]. Whilst both showed a reduction in airway smooth muscle with treatment, they were unable to correlate the degree of reduction with the number of activations applied. This difference from our study may well be explained by the wide variation observed between biopsies when measuring airway smooth muscle mass – this measurement error would serve to weaken any potential correlation with activations in a small study.

We believe that there is likely to be a minimum treatment level, below which therapy becomes less effective. The regression equation can be used to roughly approximate that in order to achieve an improvement in ACQ-5 of 0.5 units or greater, a target of 140 activations or more need to be delivered across the three treatment sessions. In this case series, 29% of activations occurred when the right lower lobe was treated, 28% when the left lower lobe was treated and 43% when the upper lobes were treated. Therefore, using the regression equation, this suggests that proceduralists should aim to deliver at least 40 activations to each of the lower lobes and 60 activations to the combined upper lobes in order to achieve an ACQ change of 0.5 units. We acknowledge that this concept requires further validation in a larger study but the recommendations do appear to be consistent with previously published guidelines [6, 15]. No correlation was observed between radiofrequency activations and change in FEV_1_, but the authors believe that this is because the magnitude of change in FEV_1_ with treatment is small (+11%) by comparison with the magnitude of change in ACQ-5 (−55%).

**Fig. 2:**
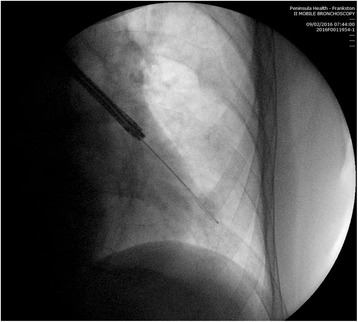
RF catheter advanced to last visible depth marking

**Fig. 3:**
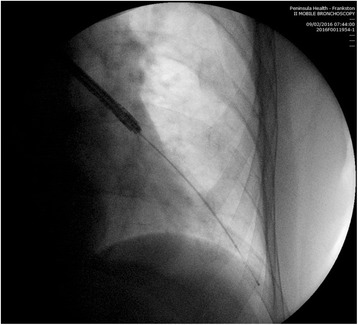
RF catheter advanced distally until resistance encountered

This study also demonstrates that, as in other areas of procedural medicine, there can be variation in technique amongst operators, and that this may affect patient outcomes. This variation only becomes apparent when comparative results are audited, as presented in Table 3. As a result of this observation, the videos taken during several procedures were reviewed and discussed by the proceduralists involved. It was concluded that the variation in activations between proceduralists was explained by variation in the distance the radiofrequency catheter was advanced into each subsegmental bronchus. The manufacturer recommends the catheter is advanced into the bronchial tree until the last depth marking on the catheter is just visible [6, 15] and a radiograph taken with the catheter at that position is shown in Fig. 2. However, it is possible to advance the catheter still further, into smaller airways until resistance is encountered, and this is demonstrated in the radiograph taken in Fig. 3. If this latter approach is adopted, this will result in a larger volume of subsegmental airways treated and higher number of activations. The authors believe that this is likely to be a common point of difference between proceduralists performing BT and may explain variation in outcomes between patients.

Our study demonstrates that there is no loss of safety in performing BT with the catheter more peripherally advanced, and that the outcomes may be superior. However, this is a small study, and only 18% of the variation in change in ACQ-5 post-treatment is explained by variation in the number of activations applied. Therefore, there may be other factors, not yet identified which predict patient response to BT. This highlights the need for a future multivariate regression analysis of predictors of response to BT in a large cohort of patients. When completed, the Bronchial Thermoplasty Global Registry [16] may provide an ideal opportunity to undertake this analysis and further examine the role of radiofrequency activations.

## Conclusion

This study demonstrates that there can be procedural differences between physicians in the application of radiofrequency treatment during BT, and that the resulting difference in activations can significantly affect patient outcomes.

## Abbreviations

ACQ-5: Asthma control questionnaire-5 item version
ERS/ATS: European Respiratory Society/American Thoracic Society
FEV_1_: Forced expiratory volume in 1 s
IQR: Interquartile range
